# Acesso à Terapia de Reperfusão e Mortalidade em Mulheres com Infarto Agudo do Miocárdio com Supradesnivelamento do Segmento ST: Registro VICTIM

**DOI:** 10.36660/abc.20190468

**Published:** 2021-04-08

**Authors:** Jussiely Cunha Oliveira, Mayse Pereira Souza Barros, Ikaro Daniel de Carvalho Barreto, Rubens Cruz Silva, Volfanio Araújo Andrade, André de Melo Oliveira, Ticiane Clair Remacre Munareto Lima, Jeferson Cunha Oliveira, Larissa Andreline Maia Arcelino, Laís Costa Souza Oliveira, Eduesley Santana-Santos, Marcos Antônio Almeida-Santos, Antônio Carlos Sousa, José Augusto Soares Barreto

**Affiliations:** 1 Universidade Federal de Sergipe Núcleo de Pós-Graduação em Ciências da Saúde São CristóvãoSE Brasil Universidade Federal de Sergipe - Núcleo de Pós-Graduação em Ciências da Saúde, São Cristóvão, SE - Brasil.; 2 Universidade Federal de Sergipe Núcleo de Pós-graduação em Enfermagem São CristóvãoSE Brasil Universidade Federal de Sergipe - Núcleo de Pós-graduação em Enfermagem, São Cristóvão, SE - Brasil.; 3 Universidade Federal de Sergipe São CristóvãoSE Brasil Universidade Federal de Sergipe – Medicina, São Cristóvão, SE - Brasil.; 4 Universidade Federal Rural de Pernambuco Núcleo de Pós-graduação em biometria e estatística aplicada RecifePE Brasil Universidade Federal Rural de Pernambuco - Núcleo de Pós-graduação em biometria e estatística aplicada, Recife, PE - Brasil.; 5 Hospital Primavera AracajuSE Brasil Hospital Primavera, Aracaju, SE - Brasil.; 6 Universidade Federal de Sergipe Hospital Universitário AracajuSE Brasil Hospital Universitário da Universidade Federal de Sergipe (HU-UFS), Aracaju, SE - Brasil.; 7 Universidade Tiradentes Programa de Pós-graduação em Saúde e Ambiente AracajuSE Brasil Programa de Pós-graduação em Saúde e Ambiente da Universidade Tiradentes, Aracaju, SE - Brasil.; 8 Fundação São Lucas Centro de Ensino e Pesquisa AracajuSE Brasil Fundação São Lucas - Centro de Ensino e Pesquisa, Aracaju, SE - Brasil.; 9 Universidade Federal de Sergipe Hospital Universitário Divisão de Cardiologia São CristóvãoSE Brasil Universidade Federal de Sergipe - Divisão de Cardiologia do Hospital Universitário, São Cristóvão, SE - Brasil.

**Keywords:** Infarto do Miocárdio, Mulheres, Reperfusão Miocárdica, Intervenção Coronária Percutânea, Morbimortalidade, Gênero e Saúde, Disparidades em Assistência à Saúde

## Abstract

**Fundamento:**

A reperfusão miocárdica é parte fundamental do tratamento para infarto agudo do miocárdio com supradesnivelamento de ST (IAMCSST) e é responsável por reduzir morbimortalidade no paciente acometido. No entanto, as taxas de reperfusão são geralmente mais baixas e as taxas de mortalidade mais altas em mulheres que em homens.

**Objetivos:**

Avaliar a prevalência do uso de terapias de reperfusão em mulheres e homens com IAMCSST nos hospitais com capacidade para realizar intervenção coronariana percutânea (ICP) no estado de Sergipe.

**Métodos:**

Trata-se de estudo transversal que utilizou dados do Registro VICTIM. Foram avaliados pacientes com diagnóstico de IAMCSST admitidos nos quatro hospitais com capacidade para realizar ICP no estado de Sergipe, sendo um público e três privados, no período de dezembro de 2014 a junho de 2018. Foi aplicada análise multivariada com modelo ajustado utilizando mortalidade como variável dependente. Em todas as análises, o nível de significância adotado foi de 5% (p<0,05).

**Resultados:**

Foram incluídos 878 voluntários com diagnóstico confirmado de IAMCSST, dos quais 33,4% eram mulheres. Apenas 53,3% dos pacientes foram submetidos à reperfusão miocárdica (134 mulheres *versus* 334 homens). A fibrinólise foi realizada somente em 2,3% de todos os pacientes (1,7% das mulheres versus 2,6% dos homens; p=0,422). Nas mulheres, a taxa de ICP primária foi menor (44% versus 54,5%; p=0,003) e a mortalidade hospitalar foi maior (16,1% versus 6,7%; p<0,001) que nos homens.

**Conclusão:**

As mulheres apresentam taxas significativamente menores de ICP primária e significativamente maiores de mortalidade hospitalar que os homens. A taxa de reperfusão em ambos os gêneros foi baixa e houve nítida subutilização de agentes trombolíticos.

## Introdução

A reperfusão miocárdica precoce constitui o pilar do tratamento do infarto agudo do miocárdio com supradesnivelamento de ST (IAMCSST) e seu uso está associado a melhores prognósticos.^1^ No entanto, em diversos locais do mundo, as mulheres têm apresentado taxas de reperfusão inferiores aos homens.^2^^–^^6^

A intervenção coronariana percutânea (ICP) atualmente é considerada o tratamento padrão ouro para o IAMCSST, por exibir melhores taxas de sucesso, maior frequência de reperfusão completa (TIMI classe 3) e menor incidência de isquemia recorrente, reinfarto e morte quando comparada com fibrinólise. O procedimento está indicado nos pacientes com IAMCSST que podem ter acesso à terapia dentro de 90 minutos do diagnóstico, além daqueles que apresentam contraindicações ao uso de fibrinolíticos ou em choque cardiogênico. Seu uso oferece benefício se realizado dentro de 12 horas do início da dor, ou até 24 horas após o diagnóstico, se houver persistência de isquemia. O uso de fibrinolíticos é de fundamental importância para os pacientes que não terão acesso em tempo hábil à ICP e no ambiente pré-hospitalar.^1^^,^^7^^,^^8^

Apesar da relevância comprovada acerca da realização de terapia de reperfusão coronariana precoce, diversos estudos têm demonstrado disparidades entre os sexos na abordagem do paciente com IAMCSST.^2^^–^^6^ As mulheres apresentam taxas de ICP e de fibrinólise inferiores aos homens,^2^^–^^6^ e mais complicações associadas à terapia de reperfusão.^9^^–^^11^ No sexo feminino, o prognóstico pós-isquemia é pior que no sexo masculino, refletindo, possivelmente, uma abordagem terapêutica menos agressiva.^4^^,^^6^^,^^12^^,^^13^

Este estudo teve por objetivo avaliar a prevalência do uso terapias de reperfusão entre mulheres e homens com IAMCSST nos hospitais com capacidade para realizar ICP no estado de Sergipe.

## Materiais e métodos

Trata-se de estudo transversal que utilizou dados do Registro VICTIM^14^ - Via Crucis para o Tratamento do Infarto do Miocárdio, coletados no período de dezembro de 2014 a junho de 2018, nos quatro hospitais de Sergipe onde a ICP é disponível. Todas as instituições são localizadas na capital, apenas uma delas atende usuários do serviço público de saúde, e é considerada de referência para o tratamento do IAMCSST. As demais instituições são privadas e oferecem atendimento sob livre demanda.

A coleta foi realizada pelos pesquisadores que utilizaram questionário próprio de pesquisa, composto pelas seguintes variáveis: idade, etnia, classe social, escolaridade, cobertura de saúde, fatores de risco, sintomas de apresentação, classificação de Killip e Kimball, escore de risco GRACE; dados referentes ao tempo do início dos sintomas à decisão de chamar socorro, tempo entre a decisão de chamar socorro à chegada ao primeiro hospital sem angioplastia, tempo de trânsito do primeiro hospital ao hospital com serviço de angioplastia e tempo total do início dos sintomas até chegada ao hospital com angioplastia; uso de tratamentos com fibrinolítico, ICP ou revascularização cirúrgica do miocárdio, além da evolução clínica dos pacientes durante a internação hospitalar após o IAM quanto à mortalidade, insuficiência cardíaca crônica, reinfarto ou choque. As informações foram coletadas por meio de entrevista com o paciente ou acompanhante e dos prontuários médicos dos pacientes.

Foram incluídos no estudo todos os pacientes maiores de 18 anos admitidos nos referidos hospitais após confirmação do IAMCSST pelo eletrocardiograma e de acordo com os critérios da V Diretriz da Sociedade Brasileira de Cardiologia,^1^ que sugere a presença de pelo menos um dos cinco critérios a seguir para que o diagnóstico de infarto seja confirmado: sintomas de isquemia miocárdica como dor no peito; alterações do segmento ST/onda T ou bloqueio completo de ramo esquerdo novos; desenvolvimento de ondas Q patológicas no ECG; perda de músculo miocárdico viável ou alteração de motilidade segmentar por exame de imagem; identificação de trombo intracoronário por angiografia ou autópsia. Além disso, os pacientes para inclusão tinham que aceitar assinar o TCLE (Termo de consentimento Livre e Esclarecido).

**Figura 1:**
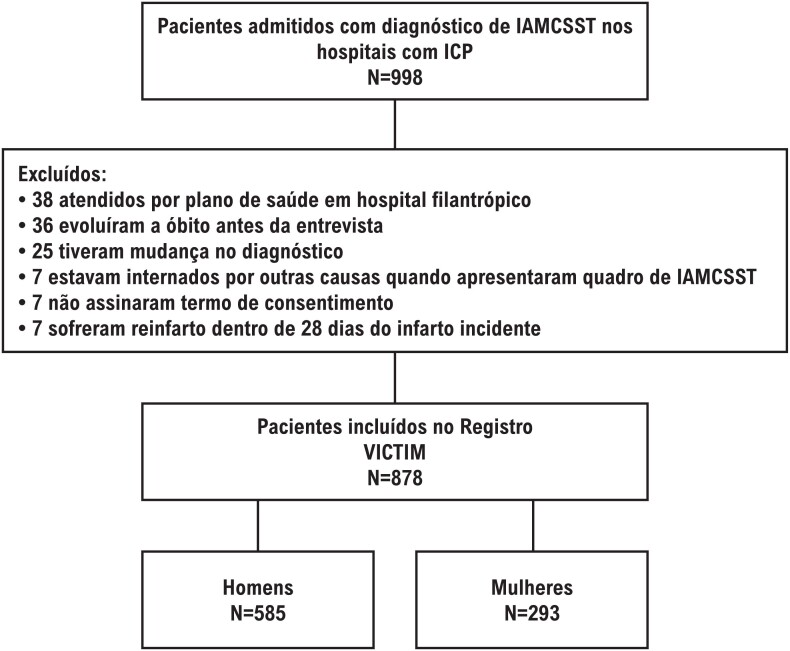
Fluxograma de pacientes excluídos; IAMCSST: infarto agudo do miocárdio com supradesnivelamento de ST.

Foram excluídos os pacientes que evoluíram para óbito antes de realizar a entrevista; que não eram elegíveis para inclusão na Via Crucis, ou seja, aqueles que se encontraram internados por outras causas quando apresentaram quadro de IAMCSST e portanto não percorreram a linha do tempo do início dos sintomas extra-hospitalar até a chegada do hospital com ICP; pacientes que não assinaram o TCLE; pacientes que sofreram reinfarto em até 28 dias do infarto incidente; e pacientes que apresentaram mudança de diagnóstico, ou seja, deram entrada nos hospitais com diagnóstico inicial de IAMCSST, mas após a realização de exames ficou constatado se tratar de outro acometimento; e aqueles atendidos por plano de saúde em hospital filantrópico (Figura 1). A coleta de dados foi feita de forma consecutiva nas instituições selecionadas.

Esta pesquisa foi aprovada pelo comitê de ética em pesquisa da Universidade Federal de Sergipe sob o parecer número 483749.

### Análise estatística

As variáveis categóricas foram descritas por frequência absoluta e relativa, e as variáveis contínuas foram descritas por média e desvio-padrão ou mediana e intervalo interquartil, conforme normalidade dos dados. Para avaliar diferenças de medidas de tendência central, primeiro aplicou-se o teste de Shapiro-Wilk para avaliar a aderência das distribuições contínuas à distribuição normal e, quando a validade desse pressuposto foi confirmada, o teste t de Student foi usado para amostras independentes; quando não, foi o utilizado o teste de Mann-Whitney. As variáveis categóricas foram avaliadas utilizando o teste de χ^2^ de Pearson. Na análise multivariada, foi utilizada a regressão logística simples, usando mortalidade como desfecho e sexo como variável independente. O modelo foi ajustado quanto à cobertura de saúde, idade, reperfusão, e escore de eisco GRACE. A análise estatística foi realizada pelo programa SPSS para Windows versão 17. Foram considerados estatisticamente significantes os resultados se os valores de p foram inferiores a 5% (p<0,05) com intervalo de confiança de 95%.

## Resultados

Foram estudados 878 pacientes com diagnóstico de IAMCSST, dos quais 33,4% eram mulheres. Comparativamente aos homens, as mulheres apresentaram-se mais idosas, a maioria faz parte da classe social E, não chegaram a concluir o nível superior, sendo que 30% delas nunca estudaram. A etnia predominante foi a não branca em ambos os grupos e o serviço mais utilizado foi o público, conforme pode ser apreciado na Tabela 1.

**Tabela 1:** Características demográficas e clínicas dos pacientes com infarto agudo do miocárdio com supradesnivelamento de ST (IAMCSST)

Demografia		Total (N=878)	Homens (N=585)	Mulheres (N=293)	p valor**	Público		p valor**	Privado		p valor**
						Homens (N=474)	Mulheres (N=250)		Homens (N=111)	Mulheres (N=43)	
**Idade, anos (média ± DP)**		61,8±12,2	61,0±11,9	63,4±12,8	0,004	61,1±12,0	62,5±12,7	0,115	60,5±11,5	68,8±12,2	<0,001
**Etnia, n (%)**											
	Branco	311 (36,2)	204 (35,7)	107 (37,4)	0,616	137 (29,5)	84 (34,6)	0,170	67 (62,0)	23 (53,5)	0,334
	Não branco	547 (63,8)	368 (64,3)	179 (62,6)		327 (70,5)	159 (65,4)		41 (38,0)	20 (46,5)	
Classe social*, **n (%)**											
	A+B	59 (7,2)	49 (8,9)	10 (3,7)	<0,001	7 (1,6)	3 (1,3)	0,006	42 (39,3)	7 (17,9)	0,049
	C+D	342 (41,6)	245 (44,5)	97 (35,7)		188 (42,4)	70 (30,0)		57 (53,3)	27 (69,2)	
	E	412 (51,2)	256 (46,5)	165 (60,7)		248 (56,0)	160 (68,7)		8 (7,5)	5 (12,8)	
**Escolaridade, n (%)**											
	Nunca estudou	217 (24,7)	129 (22,1)	88 (30,0)	0,012	125 (26,4)	83 (33,2)	0,119	4 (3,6)	5 (11,6)	0,034
	Nível fundamental ao médio	581 (66,2)	395 (67,5)	186 (63,5)		337 (71,1)	159 (63,6)		58 (52,3)	27 (62,8)	
	Nível superior	80 (9,1)	61 (10,4)	19 (6,5)		12 (2,5)	8 (3,2)		49 (44,1)	11 (25,6)	
**Cobertura de saúde, n (%)**											
	Público	724 (82,5)	474 (81,0)	250 (85,3)	0,114						
	Privado	154 (17,5)	111 (19,0)	43 (14,7)							
**Fatores de risco, n (%)**											
Diabetes mellitus		2909 (33,0)	167 (28,5)	123 (42,0)	<0,001	133 (28,1)	103 (41,2)	<0,001	34 (30,6)	20 (46,5)	0,064
Hipertensão arterial sistêmica		565 (64,4)	345 (59,0)	220 (75,1)	<0,001	271 (57,2)	183 (73,2)	<0,001	74 (66,7)	37 (86,0)	0,016
Dislipidemia		342 (39,0)	195 (33,3)	147 (50,2)	<0,001	139 (29,3)	120 (48,0)	<0,001	56 (50,5)	27 (62,8)	0,168
Tabagismo		271 (30,9)	184 (31,5)	87 (29,7)	0,594	172 (36,3)	82 (32,8)	0,350	12 (10,8)	5 (11,6)	0,885
**Número de fatores de risco, n(%)**											
	0	105 (12,0)	86 (14,7)	19 (6,5)	<0,001	70 (14,8)	17 (6,8)	<0,001	16 (14,4)	2 (4,7)	0,018
	1	277 (31,5)	208 (35,6)	69 (23,5)		173 (36,5)	63 (25,2)		35 (31,5)	6 (14,0)	
	2	320 (36,4)	202 (34,5)	118 (40,3)		162 (34,2)	96 (38,4)		40 (36,0)	22 (51,2)	
	3 ou mais	176 (20,0)	89 (15,2)	87 (29,7)		69 (14,6)	74 (29,6)		20 (18,0)	13 (30,2)	
**Sintomas de apresentação, n(%)**											
Dor típica		766 (87,2)	515 (88,0)	251 (85,7)	0,321	423 (89,2)	220 (88,0)	0,615	92 (82,9)	31 (72,1)	0,134
Dor atípica		81 (9,2)	52 (8,9)	29 (9,9)	0,626	38 (8,0)	23 (9,2)	0,586	14 (12,6)	6 (14,0)	0,824
**Classificação de KILLIPeE KIMBALL, n(%)**											
I		735 (84,5)	505 (86,9)	230 (79,6)	0,018	407 (86,0)	198 (80,2)	0,129	98 (90,7)	32 (76,2)	0,066
II		102 (11,7)	57 (9,8)	45 (15,6)		52 (11,0)	38 (15,4)		5 (4,6)	7 (16,7)	
III		19 (2,2)	9 (1,5)	10 (3,5)		7 (1,5)	8 (3,2)		2 (1,9)	2 (4,8)	
IV		14 (1,6)	10 (1,7)	4 (1,4)		7 (1,5)	3 (1,2)		3 (2,8)	1 (2,4)	
**Escore de risco ESCORE dE risco GRACE, n(%)**											
≤140 (baixo risco)		400 (48,3)	269 (49,0)	131 (47,0)	0,578	223 (50,6)	155 (48,3)	0,576	46 (42,6)	16 (39,0)	0,693
>140 (alto risco)		428 (51,7)	280 (51,0)	148 (53,0)		218 (49,4)	123 (51,7)		62 (57,4)	25 (61,0)	

* Classe social (Instituto Brasileiro de Geografia e Estatística) – A: > 20 salários mínimos, B: 10-20 salários mínimos, C: 4-10 salários mínimos, D: 2-4 salários mínimos, E: ≤ 2 salários mínimos.

** homens vs. mulheres

Acerca dos aspectos clínicos, observou-se que as mulheres, comparativamente aos homens, apresentaram maiores taxas de diabetes mellitus (42% vs 28,5%, p<0,001), hipertensão arterial sistêmica (75,1% vs 59%, p<0,001) e dislipidemia (50,2% vs 33,3%, p<0,001) do que os homens. Quanto ao número de fatores de risco, a maioria dos homens apresentam apenas um fator, porém para a associação de <3 e ≥3 fatores de risco as mulheres apresentaram maiores porcentagens. Tais taxas também se mantiveram com valores mais altos entre as mulheres quando avaliados os gêneros nos serviços públicos e privados (Tabela 1). No que tange à apresentação clínica, verifica-se que a maioria dos pacientes apresentou dor típica (88 % vs 85,7%, p=0,321), Classificação de Killip e Kimball I (86,9% VS 79,6%, p=0,018) e predominância alto risco segundo o Escore de Risco GRACE (51% vs 53%, p=0,578), em ambos os gêneros masculino e feminino respectivamente, conforme visto na Tabela 1.

Com relação ao tempo entre início de sintomas e chegada ao hospital com serviço de ICP, não houve diferença significativa entre os sexos no tempo gasto desde o início dos sintomas até a decisão de acionar ajuda médica, nem no tempo entre a decisão de chamar assistência médica e chegada ao primeiro hospital sem capacidade para realização de ICP. No entanto, o tempo gasto desde a chegada ao primeiro hospital até a chegada ao hospital com disponibilidade de ICP foi significativamente maior entre as mulheres que em homens, com mediana de 460h (IQ 233,75-1283,25) e 390h (IQ 215-775), respectivamente. O mesmo foi observado quando somente usuários do Sistema Único de Saúde foram analisados, com mediana de 535h (IQ330-1565) e 450h (IQ300-1035) entre mulheres e homens, respectivamente. Quando analisado o tempo total gasto entre o início dos sintomas até a chegada ao hospital com disponibilidade para ICP, observou-se um atraso expressivo para realização da ICP tanto em homens 545h [(IQ332-1122)] como em mulheres 705h [(IQ 71- 1612,5)]. Esse fato estava claramente associado ao tipo de sistema de saúde, uma vez que um tempo mais longo foi observado para usuários do sistema público em comparação aos de serviço privado [792,5h (456,75-1800) vs. 598h (390-1331,75)]. No serviço público, o número de mulheres que não realizaram reperfusão foi significativamente maior que no serviço privado. Não houve diferenças significativas entres os sexos quanto ao uso de fibrinolítico, sucesso da ICP e revascularização cirúrgica (Tabela 2).

**Tabela 2:** Acesso ao serviço de angioplastia, tratamento e desfechos hospitalar dos pacientes com infarto agudo do miocárdio com supradesnivelamento de ST (IAMCSST)

Linha temporal	Total (N=878)	Homens (N=585)	Mulheres (N=293)	p valor**	Público		p valor**	Privado		p valor**
					Homens (N=474)	Mulheres (N=250)		Homens (N=111)	Mulheres (N=43)	
Tempo do início dos sintomas à decisão de chamar assistência médica, h (Mediana, IIQ)	30 (13,75-150)	30 (15-160)	30 (10-150)	0,747	30 (15-150)	30 (10-131,25)	0,705	60 (15-210)	50 (15-180)	0,846
Tempo da decisão de chamar assistência médica à chegada ao primeiro hospital sem angioplastia, h (mediana, IIQ)	30 (15-60)	30 (15-60)	30 (15-60)	0,535	30 (20-60)	30 (19-60)	0,611	10 (0-30)	0 (0-16,25)	0,075
Tempo da chegada ao primeiro hospital à chegada ao hospital com serviço de angioplastia, h (mediana, IIQ)	412 (225-940)	390 (215-775)	460 (233,75-1283,25)	0,024	450 (300-1035)	535 (330-1565)	0,024	60 (30-200)	60 (30-135)	0,524
Tempo do início dos sintomas à chegada ao hospital com angioplastia, h (mediana, IIQ)	574,5 (347,75-1292,5)	545 (332-1122)	705 (371-1612,5)	0,005	598 (390-1331,75)	792,5 (456,75-1800)	0,003	221 (60-550)	150 (80-414)	0,939
Tratamento										
Fibrinolítico, n (%)	20 (2,3)	15 (2,6)	5 (1,7)	0,422	14 (3,0)	4 (1,6)	0,266	1 (0,9)	1 (2,3)	0,484
ICP* primária, n (%)	448 (51,0)	319 (54,5)	129 (44,0)	0,003	234 (49,4)	95 (38,0)	0,003	85 (76,6)	34 (79,1)	0,740
Sucesso	321 (92,8)	226 (92,6)	95 (93,1)	0,866	153 (91,1)	67 (91,8)	0,858	73 (96,1)	28 (96,6)	0,905
Revascularização cirúrgica, n (%)	29 (3,3)	20 (3,4)	9 (3,1)	0,786	14 (3,0)	7 (2,8)	0,907	6 (5,4)	2 (4,7)	0,850
Não reperfundidos, n (%)†	410 (46,7)	251 (42,9)	159 (54,3)	0,001	226 (47,7)	151 (60,4)	0,001	25 (22,5)	8 (18,6)	0,595
Desfecho hospitalar										
Mortalidade, n (%)	86 (9,8)	39 (6,7)	47 (16,1)	<0,001	37 (7,8)	42 (16,9)	<0,001	2 (1,8)	5 (11,6)	0,009
ICC, n (%)	110 (12,5)	60 (10,3)	50 (17,1)	0,004	51 (10,8)	42 (16,9)	0,020	9 (8,1)	8 (18,6)	0,062
Reinfarto, n (%)	17 (1,9)	10 (1,7)	7 (2,4)	0,486	9 (1,9)	5 (2,0)	0,919	1 (0,9)	2 (4,7)	0,131
Choque, n (%)	46 (5,2)	27 (4,6)	19 (6,5)	0,236	19 (4,0)	16 (6,4)	0,150	8 (7,2)	3 (7,0)	0,960

* ICP: Intervenção Coronariana Percutânea.

† Não reperfundidos - aqueles que não fizeram uso de fibrinolítico e ICP primária. IIQ – Intervalo Interquartil, ICC: insuficiência cardíaca crônica.

** homens vs. mulheres

Quanto aos desfechos, as mulheres apresentaram maiores taxas de mortalidade hospitalar e de insuficiência cardíaca congestiva do que os homens tanto quanto avaliado a população geral, quanto somente os usuários do SUS. Não se observou diferenças apreciáveis entre os sexos quanto à incidência de re-infarto e de choque cardiogênico (Tabela 2).

Porém, a regressão logística entre mortalidade e sexo revelou maior probabilidade de óbito para o sexo feminino [RC=2,54 (IC95%: 1,58-4,06); p<0,001], bem como quando o modelo foi ajustado para cobertura de saúde [RC=2,47 (IC95%: 1,54-3,96); p<0,001], cobertura de saúde e idade [RC=2,27 (IC95%: 1,40-3,59); p=0,001], cobertura de saúde, idade e reperfusão [RC=2,20 (IC95%: 1,35-3,59); p=0,002] cobertura de saúde, idade, reperfusão e escore de risco GRACE [RC=2,36 (IC95%: 1,44-3,88); p=0,001].

## Discussão

No presente estudo, foi observado que as mulheres apresentaram menor taxa de reperfusão e maior taxa de mortalidade que os homens. Adicionalmente, a taxa de uso de terapia de reperfusão foi baixa em ambos os sexos, e significativamente menor entre as mulheres. Diversos estudos nacionais e internacionais chamam atenção para as baixas taxas de reperfusão como um problema crescente, que necessita de estratégias urgentes mais eficazes para a implementação dos protocolos assistenciais para tratamento do IAMCSST.^15^^,^^16^

Nossos resultados são semelhantes aos já encontrados em estudos anteriores realizados nas regiões norte e nordeste do Brasil que relataram uma taxa de reperfusão em pacientes com IAMCSST de 52,5%.^16^ Isso reafirma que estamos longe de alcançar os níveis preconizados de reperfusão, tal como ocorre em países desenvolvidos, a exemplo do estudo *STRategical Reperfusion Early After Myocardial infarction* (STREAM) que observou taxas de 98,2% dos pacientes foram tratados e receberam alguma estratégia de reperfusão (trombólise com ou sem resgate, ou ICP primária).^17^

Além disso, o presente estudo revelou que existe uma desigualdade entre os sexos, com menores taxas de reperfusão em mulheres quando comparadas aos homens, fato que se intensifica quando a análise é feita somente com os usuários do SUS. Essa desigualdade tem sido verificada também em diversos estudos nacionais e internacionais,^2^^,^^3^^,^^4^^,^^6^^,^^18^ a exemplo do estudo desenvolvido na China, *Insights From the China Patient-Centered Evalueted Assessment os Cardiac Events* (PEACE), em que as chinesas apresentaram menor taxa de reperfusão mesmo quando se apresentam prontamente para o tratamento.^6^ Já o estudo intitulado *Variation in Recovery: Role of Gender on Outcomes of Young AMI Patients* (VIRGO), por sua vez, constatou que, nos Estados Unidos, as mulheres tiveram 2,31 vezes mais chances de não receber reperfusão do que os homens.^18^

Alguns estudos têm apontado que o maior número de comorbidades e o fato de apresentarem-se com quadro mais grave no momento do diagnóstico de IAMCSST poderiam expôr as mulheres ao paradoxo risco-tratamento, no qual observa-se que pacientes com maior gravidade recebem menos intervenção terapêutica.^19^^,^^20^ Nesses casos, o médico pode não oferecer tratamento adequado por julgar que a intervenção seria inútil diante da gravidade do paciente, ou por temer que os efeitos adversos superassem os benefícios gerados pela intervenção no paciente com múltiplas comorbidades.^19^ No estudo PEACE, as mulheres apresentaram mais fatores de risco que os homens, incluindo os avaliados no presente estudo, exceto tabagismo, que foi mais prevalente nos homens.^6^ A partir dessa perspectiva, o estudo *Global Registry of Acute Coronary Events* relatou que as mulheres eram mais velhas e apresentavam mais comorbidades quanto tratadas com ICP.^20^ No presente estudo, as mulheres eram mais velhas e também apresentaram um maior número de fatores de risco associados e classificação de Classificação de Killip e Kimball mais grave em comparação aos homens.

**Figura 2:**
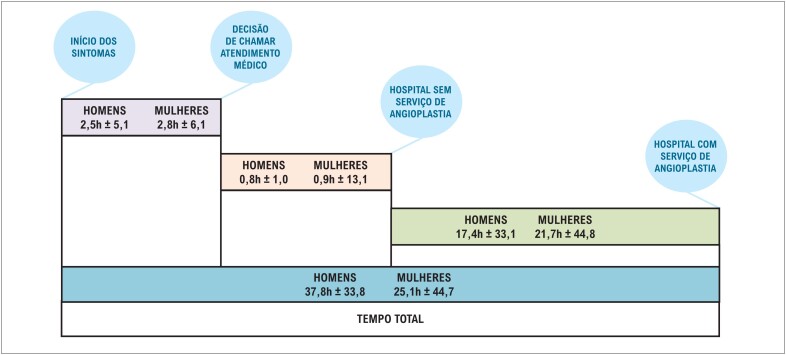
Linha temporal para o acesso de pacientes com infarto agudo do miocárdio com supradesnivelamento de ST.

Quanto ao tempo médio gasto entre a chegada ao primeiro hospital e o acesso ao hospital com serviço de ICP, observou-se no presente estudo um tempo muito superior ao sugerido pela diretriz brasileira^1^ quando analisada a população total. Ao avaliar o tempo médio total gasto do início sintomas até a chegada ao hospital com hemodinâmica estratificando a amostra por sexo, o atraso é ainda maior entre as mulheres, fato que se permanece quando analisado somente os usuários do SUS. Assim, o atraso na chegada ao hospital com angioplastia refletiu em baixas taxas de uso de ICP primária na população geral, com taxas mais baixas nas mulheres quando comparadas aos homens na população em geral (Figura 2) e entre os usuários do SUS. Já na avaliação dos usuários do sistema de saúde privado, observaram-se valores mais expressivos para realização de ICP primária no sexo feminino. No Brasil, fatores associados ao serviço de saúde, como acesso difícil e pouca estrutura, além de escolha inadequada do transporte feita pelo paciente, podem contribuir para o acesso inadequado à terapêutica, o que provoca grandes atrasos.^15^^,^^21^ Ao contrário de outros estudos,^12,22,23^ no presente estudo. As mulheres não apresentaram atrasos significativos, em comparação aos homens, na tomada de decisão de chamar por assistência médica.

Os valores encontrados para o uso do fibrinolítico foram inferiores ao observado no estudo PEACE, que constatou que em 2011, 26,8% das mulheres e 33,5% dos homens com IAMCSST foram submetidos à fibrinólise.^6^ Ademais, o estudo intitulado *Global Utilization of Streptokinase and Tissue Plasminogen Activator for Occluded Coronary Arteries* (GUSTO I) relatou uma maior taxa de mortalidade e de complicações entre mulheres após fibrinólise, quando comparadas com as voluntárias submetidas à ICP,^11^^,^^24^ visto que o uso precoce de trombolítico, quando bem indicado, reduz a mortalidade em ambos os sexos.^7^ No entanto, existem diversas barreiras ao uso desta terapia, uma vez que as mulheres apresentam mais contra indicações ao método e maiores riscos de complicações com sua utilização.^11^

Em um estudo americano realizado em 2018, a mortalidade dentro de 30 dias pós-IAMCSST no sexo feminino foi de 10,7% e de 4,6% no sexo masculino (p = 0,002).^25^ No presente estudo, as mulheres apresentaram taxas de mortalidade hospitalar e de insuficiência cardíaca pós-isquemia significativamente maiores que os homens. Os registros GUSTO I^24^ e ACC-NCDR^9^ (*National Cardiovascular Data Registry- American College of Cardiology*) corroboram a informação e mostram que as mulheres estão mais propensas a evoluírem com insuficiência cardíaca secundária a IAM. Contudo, a associação dos fatores de risco, maior demora para apresentar-se ao hospital com serviço de ICP, e idade de manifestação da doença também pode ter impactado na maior taxa de mortalidade,^11^^,^^13^ além do maior tempo de tratamento^9^^,^^14^^,^^15^^,^^26^ e menor acesso ao tratamento adequado.^4^^,^^6^^,^^12^^,^^13^^,^^15^

O presente estudo trouxe a avaliação entre os serviços públicos e privados, que revelou piores resultados para os usuários do serviço público, principalmente entre as mulheres. Ainda, nossos achados apontam para o fato de que não há políticas públicas sobre o acesso de pacientes com IAMCSST a um tratamento adequado.

### Limitações

O presente estudo apresenta algumas limitações que incluem o baixo nível social e educacional dos participantes, especialmente entre os usuários do SUS, que podem ter comprometido o autorrelato do histórico médico. A coleta do tempo porta-balão foi comprometida pela falta de registro dos tempos nos prontuários, especialmente no serviço público. Além disso, foram estudados somente mortalidade e desfechos hospitalares, e não houve acompanhamento após a alta para avaliar se houve disparidades entre os gêneros no tocante ao prognóstico após a internação hospitalar.

## Conclusão

Observou-se no presente estudo disparidades entre os gêneros, com menor taxa de ICP primária e maior mortalidade hospitalar entre as mulheres. A baixa utilização da ICP primária foi provavelmente uma das variáveis responsável pela maior mortalidade nas mulheres. As baixas taxas de reperfusão nas mulheres, tanto na população em geral como somente nos usuários do SUS, foram diretamente associadas a um atraso na chegada ao hospital com serviço de hemodinâmica, visto que a reperfusão precoce é o ponto chave do tratamento. Tais achados apontam a necessidade de estratégias de melhorias no acesso das mulheres portadoras de IAMCSST a estratégias eficazes de tratamento.
