# Supplementary material for: Acesso à Terapia de Reperfusão e Mortalidade em Mulheres com Infarto Agudo do Miocárdio com Supradesnivelamento do Segmento ST: Registro VICTIM
Source: Arq Bras Cardiol. 2021 Apr 8;116(4):695–703. [Article in Portuguese] doi: 10.36660/abc.20190468 (PMC8121383; doi:10.36660/abc.20190468)
Supplement: Translation

# Translation: Access to Reperfusion Therapy and Mortality in Women with ST-Segment–Elevation Myocardial Infarction: VICTIM Register

**Authors:** Jussiely Cunha Oliveira, Mayse Pereira Souza Barros, Ikaro Daniel de Carvalho Barreto, Rubens Cruz Silva, Volfanio Araújo Andrade, André de Melo Oliveira, Ticiane Clair Remacre Munareto Lima, Jeferson Cunha Oliveira, Larissa Andreline Maia Arcelino, Laís Costa Souza Oliveira, Eduesley Santana-Santos, Marcos Antônio Almeida-Santos, Antônio Carlos Sousa, José Augusto Soares Barreto

**Affiliations:** 1 São CristóvãoSE Brazil Universidade Federal de Sergipe - Núcleo de Pós-Graduação em Ciências da Saúde, São Cristóvão, SE - Brazil.; 2 São CristóvãoSE Brazil Universidade Federal de Sergipe - Núcleo de Pós-graduação em Enfermagem, São Cristóvão, SE - Brazil.; 3 São CristóvãoSE Brazil Universidade Federal de Sergipe – Medicina, São Cristóvão, SE - Brazil.; 4 RecifePE Brazil Universidade Federal Rural de Pernambuco - Núcleo de Pós-graduação em biometria e estatística aplicada, Recife, PE - Brazil.; 5 AracajuSE Brazil Hospital Primavera, Aracaju, SE - Brazil.; 6 AracajuSE Brazil Hospital Universitário da Universidade Federal de Sergipe (HU-UFS), Aracaju, SE - Brazil.; 7 AracajuSE Brazil Programa de Pós-graduação em Saúde e Ambiente da Universidade Tiradentes, Aracaju, SE - Brazil.; 8 AracajuSE Brazil Fundação São Lucas - Centro de Ensino e Pesquisa, Aracaju, SE - Brazil.; 9 São CristóvãoSE Brazil Universidade Federal de Sergipe - Divisão de Cardiologia do Hospital Universitário, São Cristóvão, SE - Brazil.

**Keywords:** Myocardial Infarction, Women, Myocardial Reperfusion, Percutaneous Coronary Intervention, Morbimortality, Gender and Health, Healthcare Disparities

## Abstract

**Background:**

Myocardial reperfusion is a fundamental part of the treatment for ST elevation myocardial infarction (STEMI) and is responsible for reducing morbidity and mortality in affected patients. However, reperfusion rates are usually lower and mortality rates higher in women compared to men.

**Objectives:**

To evaluate the prevalence of the use of reperfusion therapies among women and men with STEMI in hospitals where percutaneous coronary intervention (PCI) is available in the state of Sergipe.

**Methods:**

This is a cross-sectional study that used data from the VICTIM Register. Patients diagnosed with STEMI admitted to the four hospitals (one public and three private) where PCI is available in the state of Sergipe were evaluated, from December 2014 to June 2018. A multivariate analysis with adjusted model using mortality as a dependent variable was made. In all analyses, the level of significance adopted was 5% (p < 0.05).

**Results:**

A total of 878 volunteers with a confirmed diagnosis of STEMI, of which 33.4% were women, were included in the study. Only 53.3% of the patients underwent myocardial reperfusion (134 women versus 334 men). Fibrinolysis was performed only in 2.3% of all patients (1.7% of women versus 2.6% of men; p = 0.422). The rate of primary PCI was lower (44% versus 54.5%; p = 0.003) and hospital mortality was higher (16.1% versus 6.7%; p < 0.001) in women than in men.

**Conclusion:**

Women have significantly lower rates of primary PCI and higher hospital mortality. Reperfusion rates were low in both sexes and there was a clear underutilization of thrombolytic agents.

## Introduction

Early myocardial reperfusion is the mainstay of the treatment of acute myocardial infarction with ST elevation (STEMI) and its use is associated with better prognosis.^1^ However, in different parts of the world, women have presented lower reperfusion rates than men.^2^^–^^6^

Percutaneous coronary intervention (PCI) is currently considered the gold standard treatment for STEMI because it has better success rates, a higher frequency of complete reperfusion (TIMI grade 3) and a lower incidence of recurrent ischemia, reinfarction and death when compared to fibrinolysis. The procedure is indicated for patients with STEMI who may have access to therapy within 90 minutes of diagnosis, in addition to those who have contraindications to the use of fibrinolytic drugs or in cardiogenic shock. Its use is beneficial if performed within 12 hours of the onset of pain, or up to 24 hours after diagnosis, if ischemia persists. The use of fibrinolytic drugs is of fundamental importance for patients who will not have timely access to PCI and patients in the prehospital environment.^1^^,^^7^^,^^8^

Despite the proven relevance of early coronary reperfusion therapy, several studies have shown disparities between sexes when it comes to the approach for patients with STEMI.^2^^–^^6^ Women have lower rates of PCI and fibrinolysis than men^2^^–^^6^ as well as more complications associated with reperfusion therapy.^9^^–^^11^ In women, the prognosis after ischemia is worse than in men, possibly reflecting a less aggressive therapeutic approach.^4^^,^^6^^,^^12^^,^^13^

This study aimed to assess the prevalence of the use of reperfusion therapies among women and men with STEMI in hospitals where PCI is available in the state of Sergipe.

## Materials and methods

This is a cross-sectional study that used data from the VICTIM Register^14^ - Via Crucis for Treatment of Myocardial Infarction, collected from December 2014 to June 2018, in the four hospitals in Sergipe where PCI is available. All institutions are located in the capital; only one of them serves public service users and is renowned for its performance in the treatment of STEMI. The other institutions are private and offer assistance on demand.

The collection was carried out by the researchers using their own research questionnaire which was composed of the following variables: age, ethnicity, social class, education, health coverage, risk factors, symptoms at presentation, Killip and Kimball classification, GRACE risk score; data regarding the time elapsed between the onset of symptoms and the decision to call for help, the decision to call for help to arrival at the first hospital without angioplasty, time from the first hospital to the hospital with angioplasty, and the total time elapsed since the onset of symptoms until arrival at the hospital with angioplasty; use of fibrinolytic treatments, PCI or coronary artery bypass grafting, in addition to clinical course of patients during hospitalization after AMI regarding mortality, chronic heart failure, re-infarction, or shock. The information was collected through interviews with the patient or caregiver and from patients’ medical records.

The study included all patients over 18 years of age admitted to the above-mentioned hospitals after confirmation of STEMI by an electrocardiogram, and according to the V Brazilian Society of Cardiology guidelines,^1^ which suggests the presence of at least one of the following five criteria for confirmation of the diagnosis of infarction: symptoms of myocardial ischemia such as chest pain; changes in the ST segment/T wave or complete left bundle branch block; development of pathological Q waves on the ECG; imaging evidence of loss of viable myocardium or wall motion abnormalities; or the identification of an intracoronary thrombus by angiography or autopsy. In addition, patients signed an informed consent form before inclusion in the study.

Patients who died before the interview, patients who were not eligible for inclusion in the Via Crucis, that is, who were hospitalized for other causes when STEMI was detected and hence did not go through the timeline from the onset of out-of-hospital symptoms until arrival at the hospital with PCI; patients who did not sign the informed consent form; who suffered reinfarction within 28 days after the incident myocardial infarction; patients who had a change in diagnosis, that is, those who were admitted for STEMI, but were identified with another problem after the exams; and patients assisted by a health plan seen in a philanthropic hospital (Figure 1) were excluded from the study. Data collection was carried out consecutively in the selected institutions.

**Figure 1:** Flowchart of excluded patients; STEMI: ST elevation myocardial infarction.

This research was approved by research ethics committee of the Federal University of Sergipe (UFS) (approval number 483,749).

### Statistical analysis

Categorical variables were described using absolute and relative frequency, and continuous variables were described using mean and standard deviation or median and interquartile range, according to the normality of the data. To assess differences in measures of central tendency, the Shapiro-Wilk test was first applied to assess the adherence of continuous variables to the normal distribution, and when the validity of this assumption was confirmed, the Student’s t test was used for independent samples; otherwise, the Mann-Whitney test was used. Categorical variables were assessed using Pearson’s X^2^ test. In the multivariate analysis, a simple logistic regression was used; mortality was the outcome and sex was the independent variable. The model was adjusted for health coverage, age, reperfusion and GRACE Risk Score. The statistical analysis was performed using the SPSS software for Windows version 17; results were considered statistically significant if p-values were less than 0.05, with 95% confidence interval.

## Results

A total of 878 patients (33.4% women) diagnosed with STEMI were studied. Compared to men, women were older, most belonged to lower social class, did not finish college, and 30% never went to school. The predominant ethnic group was non-white in both groups and the most used service was the public service, as can be seen in Table 1.

**Table 1:** Demographic and clinical characteristics of patients with ST elevation myocardial infarction (STEMI)

Demography		Total (N = 878)	Men (N = 585)	Women (N = 293)	p value**	Public		p value**	Private		p value**
						Men (N = 474)	Women (N = 250)		Men (N = 111)	Women (N = 43)	
Age, years (mean ± SD)		61.8±12.2	61.0±11.9	63.4±12.8	0.004	61.1±12.0	62.5±12.7	0.115	60.5±11.5	68.8±12.2	<0.001
**Ethnicity, n (%)**											
	White	311 (36.2)	204 (35.7)	107 (37.4)	0.616	137 (29.5)	84 (34.6)	0.170	67 (62.0)	23 (53.5)	0.334
	Non-white	547 (63.8)	368 (64.3)	179 (62.6)		327 (70.5)	159 (65.4)		41 (38.0)	20 (46.5)	
Social class*, n (%)											
	A + B	59 (7.2)	49 (8.9)	10 (3.7)	<0.001	7 (1.6)	3 (1.3)	0.006	42 (39.3)	7 (17.9)	0.049
	C + D	342 (41.6)	245 (44.5)	97 (35.7)		188 (42.4)	70 (30.0)		57 (53.3)	27 (69.2)	
	E	412 (51.2)	256 (46.5)	165 (60.7)		248 (56.0)	160 (68.7)		8 (7.5)	5 (12.8)	
**Schooling, n (%)**											
	Never went to school	217 (24.7)	129 (22.1)	88 (30.0)	0.012	125 (26.4)	83 (33.2)	0.119	4 (3.6)	5 (11.6)	0.034
	Primary to secondary school	581 (66.2)	395 (67.5)	186 (63.5)		337 (71.1)	159 (63.6)		58 (52.3)	27 (62.8)	
	Higher education	80 (9.1)	61 (10.4)	19 (6.5)		12 (2.5)	8 (3.2)		49 (44.1)	11 (25.6)	
**Health coverage, n (%)**											
	Public	724 (82.5)	474 (81.0)	250 (85.3)	0.114						
	Private	154 (17.5)	111 (19.0)	43 (14.7)							
**Risk factors, n (%)**											
Diabetes mellitus		2909 (33.0)	167 (28.5)	123 (42.0)	<0.001	133 (28.1)	103 (41.2)	<0.001	34 (30.6)	20 (46.5)	0.064
Systemic arterial hypertension		565 (64.4)	345 (59.0)	220 (75.1)	<0.001	271 (57.2)	183 (73.2)	<0.001	74 (66.7)	37 (86.0)	0.016
Dyslipidemia		342 (39.0)	195 (33.3)	147 (50.2)	<0.001	139 (29.3)	120 (48.0)	<0.001	56 (50.5)	27 (62.8)	0.168
Smoking		271 (30.9)	184 (31.5)	87 (29.7)	0.594	172 (36.3)	82 (32.8)	0.350	12 (10.8)	5 (11.6)	0.885
**Number of risk factors, n (%)**											
	0	105 (12.0)	86 (14.7)	19 (6.5)	<0.001	70 (14.8)	17 (6.8)	<0.001	16 (14.4)	2 (4.7)	0.018
	1	277 (31.5)	208 (35.6)	69 (23.5)		173 (36.5)	63 (25.2)		35 (31.5)	6 (14.0)	
	2	320 (36.4)	202 (34.5)	118 (40.3)		162 (34.2)	96 (38.4)		40 (36.0)	22 (51.2)	
	3 or more	176 (20.0)	89 (15.2)	87 (29.7)		69 (14.6)	74 (29.6)		20 (18.0)	13 (30.2)	
**Presentation symptoms, n (%)**											
Typical pain		766 (87.2)	515 (88.0)	251 (85.7)	0.321	423 (89.2)	220 (88.0)	0.615	92 (82.9)	31 (72.1)	0.134
Atypical pain		81 (9.2)	52 (8.9)	29 (9.9)	0.626	38 (8.0)	23 (9.2)	0.586	14 (12.6)	6 (14.0)	0.824
**KILLIP AND KIMBALL CLASSIFICATION, n (%)**											
I		735 (84.5)	505 (86.9)	230 (79.6)	0.018	407 (86.0)	198 (80.2)	0.129	98 (90.7)	32 (76.2)	0.066
II		102 (11.7)	57 (9.8)	45 (15.6)		52 (11.0)	38 (15.4)		5 (4.6)	7 (16.7)	
III		19 (2.2)	9 (1.5)	10 (3.5)		7 (1.5)	8 (3.2)		2 (1.9)	2 (4.8)	
IV		14 (1.6)	10 (1.7)	4 (1.4)		7 (1.5)	3 (1.2)		3 (2.8)	1 (2.4)	
**GRACE RISK SCORE, n (%)**											
≤ 140 (low risk)		400 (48.3)	269 (49.0)	131 (47.0)	0.578	223 (50.6)	155 (48.3)	0.576	46 (42.6)	16 (39.0)	0.693
> 140 (high risk		428 (51.7)	280 (51.0)	148 (53.0)		218 (49.4)	123 (51.7)		62 (57.4)	25 (61.0)	

* Social class (IBGE) - A: > 20 minimum wages, B: 10-20 minimum wages, C: 4-10 minimum wages, D: 2-4 minimum wages, E: ≤ 2 minimum wages.

** men vs. women.

Regarding the time from symptom onset to arrival at the hospital where PCI was available, no significant difference between sexes was observed in the time spent from the onset of symptoms to the decision to call for medical help, or in the time between the decision to call medical help and arrival at the first hospital without capacity to perform PCI. However, the time spent from presentation at the first hospital to arrival at the hospital where PCI was available was significantly longer for women than men, with a median of 460 h (IQ 233.75-1283.25) and 390 h (IQ 215- 775), respectively. The same was observed when only users of the Unified Health System (SUS) were analyzed, with a median of 535h (IQ 330-1565) and 450h (IQ 300-1035) for women and men, respectively. As for the total time spent between the onset of symptoms and arrival at the hospital where PCI was available, there was a significant delay to treatment in both men [545h (IQ332-1122)] and women 705h [(IQ 71-1612.5)]. This was clearly associated with the type of health system, as the time was longer for users of the public system compared with users of the private one [792.5h (456.75-1800) and 598h (390-1331.75), respectively]. In addition, in the public service, the number of women who were not reperfused was significantly greater than in the private service. No differences were found in the use of fibrinolytic agents, success of PCI, and coronary artery bypass grafting between men and women (Table 2).

**Table 2:** Access to the angioplasty service, treatment and hospital outcomes of patients with ST elevation myocardial infarction (STEMI)

Timeline	Total (N = 878)	Men (N = 585)	Women (N = 293)	p value**	Public		p value**	Private		p value**
					Men (N = 474)	Women (N = 250)		Men (N = 111)	Women (N = 43)	
Time from symptom onset to decision to call for help, h (median, IIQ)	30 (13.75-150)	30 (15-160)	30 (10-150)	0.747	30 (15-150)	30 (10-131.25)	0.705	60 (15-210)	50 (15-180)	0.846
Time from decision to call for help to arrival at the first hospital, h (median, IIQ)	30 (15-60)	30 (15-60)	30 (15-60)	0.535	30 (20-60)	30 (19-60)	0.611	10 (0-30)	0 (0-16.25)	0.075
Time from arrival at the first hospital to arrival at the hospital with angioplasty service, h (median, IIQ)	412 (225-940)	390 (215-775)	460 (233.75-1283.25)	0.024	450 (300-1035)	535 (330-1565)	0.024	60 (30-200)	60 (30-135)	0.524
Time from symptom onset to arrival at the hospital with angioplasty, h (median, IIQ)	574.5 (347.75-1292.5)	545 (332-1122)	705 (371-1612.5)	0.005	598 (390-1331.75)	792.5 (456.75-1800)	0.003	221 (60-550)	150 (80-414)	0.939
**Treatment**										
Fibrinolytic agent, n (%)	20 (2.3)	15 (2.6)	5 (1.7)	0.422	14 (3.0)	4 (1.6)	0.266	1 (0.9)	1 (2.3)	0.484
Primary PCI*, n (%)	448 (51.0)	319 (54.5)	129 (44.0)	0.003	234 (49.4)	95 (38.0)	0.003	85 (76.6)	34 (79.1)	0.740
Success	321 (92.8)	226 (92.6)	95 (93.1)	0.866	153 (91.1)	67 (91.8)	0.858	73 (96.1)	28 (96.6)	0.905
Coronary artery bypass grafting, n (%)	29 (3.3)	20 (3.4)	9 (3.1)	0.786	14 (3.0)	7 (2.8)	0.907	6 (5.4)	2 (4.7)	0.850
Not reperfused, n (%) †	410 (46.7)	251 (42.9)	159 (54.3)	0.001	226 (47.7)	151 (60.4)	0.001	25 (22.5)	8 (18.6)	0.595
**Hospital outcome**										
Mortality, n (%)	86 (9.8)	39 (6.7)	47 (16.1)	<0.001	37 (7.8)	42 (16.9)	<0.001	2 (1.8)	5 (11.6)	0.009
CHF, n (%)	110 (12.5)	60 (10.3)	50 (17.1)	0.004	51 (10.8)	42 (16.9)	0.020	9 (8.1)	8 (18.6)	0.062
Reinfarction, n (%)	17 (1.9)	10 (1.7)	7 (2.4)	0.486	9 (1.9)	5 (2.0)	0.919	1 (0.9)	2 (4.7)	0.131
Shock, n (%)	46 (5.2)	27 (4.6)	19 (6.5)	0.236	19 (4.0)	16 (6.4)	0.150	8 (7.2)	3 (7.0)	0.960

* PCI: Percutaneous Coronary Intervention.

† Not reperfused - those who did not use fibrinolytic agent and primary PCI. IIQ - Interquartile Interval; CHF: chronic heart failure;

** men vs. women

However, the logistic regression between mortality and sex revealed a higher likelihood of death in the female sex [CR = 2.54 (95% CI: 1.58-4.06); p < 0.001], as well as when adjusted for health coverage [CR = 2.47 (95% CI: 1.54-3.96); p < 0.001], health coverage and age [CR = 2.27 (95% CI: 1.40-3.59); p = 0.001], health coverage, age and reperfusion [CR = 2.20 (95% CI: 1.35-3.59); p = 0.002], health coverage, age, reperfusion and GRACE risk score [CR = 2.36 (95% CI: 1.44-3.88); p = 0.001].

## Discussion

In the present study, lower reperfusion rates and higher mortality rates were observed in women than in men. Moreover, the rate of use of reperfusion therapy was low in both sexes, and significantly lower in women. Several national and international studies have called attention to the low rates of reperfusion as a growing problem, and thus more effective strategies for the implementation of care protocols for the treatment of STEMI are urgently required.^15^^,^^16^

The present findings are similar to those of previous studies carried out in the north and northeast of Brazil reporting a reperfusion rate in patients with STEMI of 52.5%.^16^ This confirms that we are far from meeting the recommendations on reperfusion rates, such as observed in developed countries. For example, the STRategical Reperfusion Early After Myocardial infarction (STREAM) study observed rates as high as 98.2% of patients treated and receiving some reperfusion strategy (thrombolysis with or without rescue or primary PCI) in a developed country.^17^

The present study also revealed an inequality between sexes, with lower rates of reperfusion in women when compared to men, especially when analyzing data from SUS users. Such inequality was also verified in several national and international studies,^2^^,^^3^^,^^4^^,^^6^^,^^18^ such as the study conducted in China – Insights From the China Patient-Centered Evaluated Assessment the Cardiac Events (PEACE) – in which Chinese women had lower reperfusion rates even when they were promptly referred for treatment.^6^ The study entitled Variation in Recovery: Role of Gender on Outcomes of Young AMI Patients (VIRGO) found that, in the United States, women were 2.31 times more likely to not receive reperfusion than men.^18^

Some studies have pointed out that the greater number of comorbidities and the fact of having a more severe condition at the time of the diagnosis of STEMI could expose women to the risk-treatment paradox, in which it is observed that patients with a more severe condition receive less therapeutic interventions.^19^^,^^20^ In these cases, the physician may not offer adequate treatment because he believes that the intervention will be useless in view of the patient’s severe state, or because he fears that the adverse effects will outweigh the benefits generated by the intervention in the patient with multiple comorbidities.^19^ In the PEACE study, women had a higher frequency of risk factors than men, including those assessed in the present study, except smoking, which was more prevalent among men.^6^ From this perspective, the Global Registry of Acute Coronary Events study found that women were older and had more comorbidities when treated with PCI.^20^ Accordingly, in the present study, women were older, and had a higher number of associated risk factors and a more severe Killip and Kimball classification than men.

As for the average time spent between arrival at the first hospital and access to the hospital with PCI service, a much longer time than that suggested by the Brazilian guideline^1^ was observed when analyzing the total population. Analysis of the average time from symptom onset to arrival at the hospital with hemodynamics, stratified by sex, treatment delay was even greater among women, which was maintained in the analysis of SUS users only. Thus, the delay to arrive at the hospital with angioplasty reflected in low rates of use of primary PCI in the general population, with lower rates in women when compared with men in the general population (Figure 2) and among SUS users. In the evaluation of users of private health services, more expressive values were observed for the performance of primary PCI in females. In Brazil, factors associated with health service, such as difficult access and little structure, besides the inadequate choice of transportation made by patients, can contribute to inadequate access to therapy, leading to long delays.^15^^,^^21^ Contrary to other reports,^12^^,^^22^^,^^23^ in the present study, women did not experience significant delays, compared to men, when making a decision to call for help.

**Figure 2:** Timeline of access to treatment of patients with ST elevation myocardial infarction.

Values found for the use of fibrinolytic agents were lower than those observed in the PEACE study, which found that in 2011, 26.8% of women and 33.5% of men with STEMI were submitted to fibrinolysis.^6^ Furthermore, the study entitled Global Utilization of Streptokinase and Tissue Plasminogen Activator for Occluded Coronary Arteries (GUSTO I) reported a higher rate of mortality and complications among women after fibrinolysis when compared with the volunteers submitted to PCI,^11^^,^^24^ since the early thrombolytic therapy, with due indication, reduces mortality in both sexes.^7^ However, there are several barriers to the use of this therapy, since women have more contraindications to this method and greater risks of complications.^11^

In an American study conducted in 2018, mortality within 30 days after STEMI was 10.7% in women and 4.6% in men (p = 0.002).^25^ In the present study, women had significantly higher rates of hospital mortality and post-ischemic heart failure than men. The GUSTO I^24^ and ACC-NCDR^9^(National Cardiovascular Data Registry- American College of Cardiology) records corroborate the information and show that women are more likely to develop heart failure following AMI. However, the association of risk factors, greater delay in reach the hospital with PCI service, and age of appearance of the condition may also have impacted the higher mortality rate,^11^^,^^13^ in addition to the longer time spent receiving treatment^9^^,^^14^^,^^15^^,^^26^ and less access to adequate treatment.^4^^,^^6^^,^^12^^,^^13^^,^^15^

The present study brought an assessment between public and private services, which revealed worse results for users of the public service, especially among women. In addition, our findings point to an absence of public policies regarding adequate access of patients with STEMI to adequate treatment.

### Limitations

The present study has some limitations that include the low social and educational level of participants, especially among SUS users, which may have compromised the self-reporting of their medical history. The collection of data on door-to-balloon time was compromised by the lack of information of times in the medical records, especially in the public service. In addition, only mortality and hospital outcomes were studied and there was no follow-up after discharge to assess whether there were disparities between sexes regarding prognosis after hospitalization.

## Conclusion

Disparities between sexes were observed in the present study with lower rates of primary PCI and higher rates of hospital mortality among women. The low use of primary PCI was probably one of the variables responsible for the higher mortality in women. The low rates of reperfusion in women, both considering the general population and in SUS users only, were directly associated with delayed arrival at the hospital with hemodynamic service, since early reperfusion is the key point of treatment. Such findings point to the need for strategies to improve access of women with STEMI to effective therapeutic strategies.
